# High-altitude retinopathy and retinal hemorrhage: A case report

**DOI:** 10.1097/MD.0000000000040780

**Published:** 2024-11-29

**Authors:** Rao Fu, Dachuan Liu, Yenan Wang

**Affiliations:** a Department of Ophthalmology, Xuanwu Hospital, Capital Medical University, Beijing, China.

**Keywords:** case report, high-altitude retinopathy, high-altitude sickness, hypoxia, retinal hemorrhage

## Abstract

**Rationale:**

High-altitude retinopathy (HAR) is a retinal disorder caused by prolonged exposure to hypoxic environments at high altitudes, posing a significant threat to visual health and potentially leading to severe vision impairment. There have been limited references of HAR, with observations of its clinical manifestations and prognosis remaining insufficient. Some reports suggest that HAR may spontaneously resolve without eliciting any noticeable symptoms. This case focuses on the disease progression and follow-up results of a typical HAR patient, and a comprehensive ophthalmic examination was conducted, encompassing not only fundus examination and optic coherence tomography (OCT), but also fundus fluorescein angiography, Humphrey visual field examination, and electroretinogram.

**Patient concerns:**

A 41-year-old healthy Chinese man ascended from an altitude of 143 ft (43.5 m) to 13,780 ft (4200 m) rapidly. After living locally for 4 months, he developed decreased vision accompanied by visual field defects in his left eye.

**Diagnoses:**

Ophthalmic examination revealed bilateral papilledema, peripapillary hemorrhage in his left eye, and retinal hemorrhage in the macular area of optic papilla. He was diagnosed with HAR.

**Interventions:**

Peribulbar injection of triamcinolone acetonide in the left eye, along with oral ginkgo biloba tablets to improve microcirculation, as well as mecobalamin tablets and vitamin B1 tablets for neural nutrition.

**Outcomes:**

After 4 months, the patient’s visual field defect decreased, and retinal hemorrhage was absorbed.

**Lessons:**

This case comprehensively reveals the clinical features of HAR, and highlights that HAR can lead to permanent visual function impairment if the individual fails to promptly leave the pathogenic environment.

## 1. Introduction

When an individual rapidly ascend from a low-altitude area to a high-altitude area exceeding 3000 m, a constellation of health conditions resulting from hypoxia are collectively referred to as altitude sickness. The clinical manifestations of high-altitude diseases include acute mountain sickness, high-altitude retinopathy (HAR), high-altitude cerebral edema (HACE) and high-altitude pulmonary edema (HAPE). Researches has conclusively demonstrated that HAR is associated with both the emergence and progression of HACE and HAPE.^[1]^

The clinical manifestations of HAR involve tortuous retinal veins, diffuse or punctate retinal hemorrhage (occasionally located in the macula), vitreous hemorrhage, papillary edema, peripapillary hemorrhage, etc.^[2]^ This case study presents a 41-year-old healthy Chinese man who ascended from an altitude of 143 ft (43.5 m) to 13,780 ft (4200 m) rapidly developing the symptoms of HAR.

There have been limited references of HAR, with observations of its clinical manifestations and prognosis remaining insufficient. Some reports suggest that HAR may spontaneously resolve without eliciting any noticeable symptoms. This case focuses on the disease progression and follow-up results of a typical HAR patient, and a comprehensive ophthalmic examination was conducted, encompassing not only fundus examination and optic coherence tomography (OCT), but also fundus fluorescein angiography (FFA), Humphrey visual field examination, and electroretinogram (ERG).

## 2. Case report

In July 2022, a 41-year-old healthy Chinese man ascended from an altitude of 143 ft (43.5 m) and to 13,780 ft (4200 m) above sea level in Yushu, Qinghai Province, within 1 day. Yushu City is located in the eastern part of the Qinghai-Tibet Plateau, which is the backbone of the terrain formed by the remaining veins of Tangura Mountain, and has an average elevation of 4493.4 m. It is a typical plateau and alpine climate. The oxygen content of the air is low, only 40% to 60% of what it is at sea level. The atmospheric pressure is about 60 kPa.^[3]^ He resided there until November 2022 for 4 months, engaging in approximately 4 to 8 hours of daily physical activity. Subsequently, he experienced reduced vision and visual field defects in his left eye, without any accompanying systemic symptoms such as headache, dizziness, vomiting, or any other notable discomfort. After resting, his symptoms persisted without any improvement, he returned to the area of an elevation of 143 ft (43.5 m) to attend an ophthalmology clinic.

**Figure 1.**
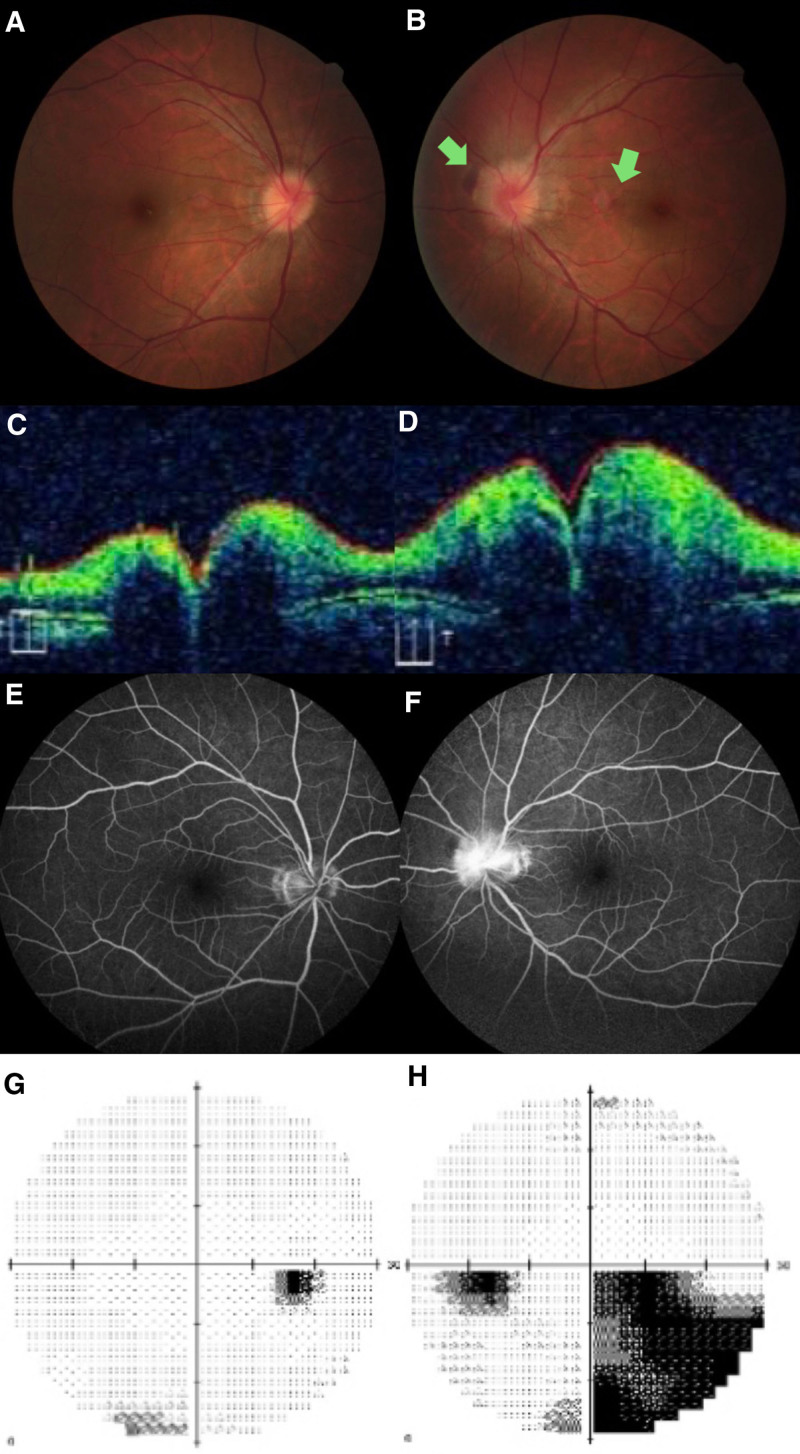
Color fundus photography showed indistinct and congested optic disc boundary, papilledema bulging, which was more severe in the left than in the right eye. The retinal veins exhibited a slight tortuosity and dilation, while the ratio of retinal arteries to veins approximated 1:2. As indicated by the arrow, lamellar superficial hemorrhage was observed on the nasal side of the left eye around the optic disc, and lamellar retinal hemorrhage was in the optic papilla macular area (A, B); OCT showed a W-shaped protuberance in the optic disc (C, D); FFA indicated mild late-stage fluorescein leakage in the right optic disc and significant late-stage fluorescein leakage in the left optic disc. Blocked fluorescence was observed on the nasal side of the optic disc in the left eye, which was consistent with color fundus hemorrhage (E,F); Humphrey’s visual field showed an inferior visual field scotoma in the right eye and an inferonasal visual field defect in the left eye (G,H). FFA = fundus fluorescein angiography, OCT = optic coherence tomography.

The patient’s chief complaint was decreased visual acuity in his left eye, accompanied by a subnasal visual field defect lasting for 2 weeks. He reported no history of other ocular diseases, systemic diseases, injuries, or surgeries. Furthermore, he was not taking any oral medications. During the initial examination, the corrected visual acuity was 1.0 (20/20) in the right eye and 0.7 (14/20) in the left eye. Intraocular pressure was 12 mm Hg in both eyes. Slit-lamp biomicroscope showed no abnormal findings in the anterior segments in both eyes. Color fundus photographs showed indistinct and congested disc boundaries with papilledema bulges, which were more severe in the left compared to the right. The retinal veins exhibited a slight tortuosity and dilation, while the ratio of retinal arteries to veins approximated 1:2. Lamellar superficial hemorrhage was observed on the nasal side of the left eye around the optic disc, and lamellar retinal hemorrhage was observed in the optic papilla macular area (Fig. 1A, B). OCT showed an increase in the thickness of the optic nerve fiber layer in both eyes and a W-shaped protuberance in the optic disc (Fig. 1C, D). FFA indicated mild late-stage fluorescein leakage in the right optic disc and significant late-stage fluorescein leakage in the left optic disc. Blocked fluorescence was observed on the nasal side of the optic disc in the left eye, which was consistent with retinal hemorrhage shown in color fundus photography (Fig. 1E, F). Humphrey’s visual field showed an inferior visual field scotoma in the right eye (MD = −1.64 dB) and an inferonasal visual field defect in the left eye (MD = −10.09 dB; Fig. 1G, H).

At the same time, the patient underwent routine blood and biochemical tests. His hematocrit and hemoglobin levels were found to be elevated. This observation indicates an elevation in blood viscosity, which is notably considered as a contributing factor to HAR.^[4]^ The patient was given a peribulbar injection of triamcinolone acetonide into the left eye for anti-inflammatory treatment, oral ginkgo biloba tablets to improve microcirculation, oral mecobalamin tablets and vitamin B1 tablets for neural nutrition.

**Figure 2.**
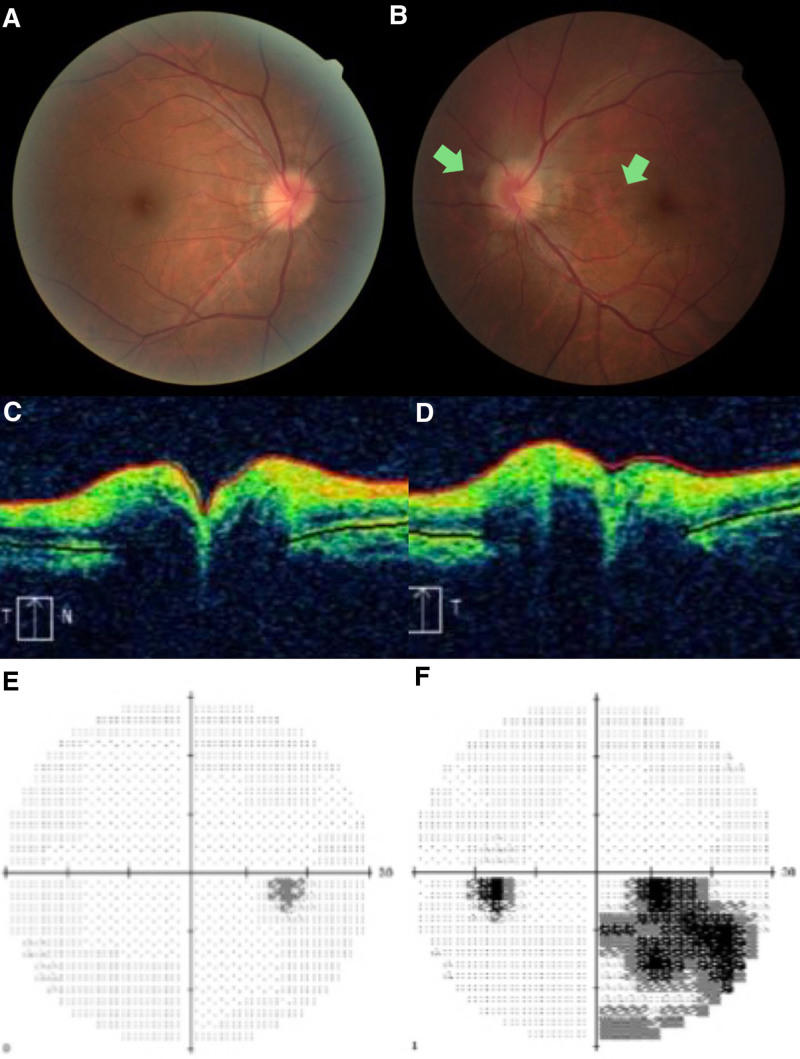
Color fundus photography revealed that the papilledema in both eyes had decreased compared to the previous examination. The patchy superficial hemorrhage in the macular area of the left eye had completely resolved, and the patchy superficial hemorrhage in the nasal side of the optic disc of the left eye had also been significantly absorbed (A, B); OCT showed a reduction in papilledema (C, D); Humphrey visual field showed that the scotoma in the right eye has disappeared, the defect in the inferior nasal quadrant of the visual field in the left eye was smaller than that before (E, F). OCT = optic coherence tomography.

**Figure 3.**
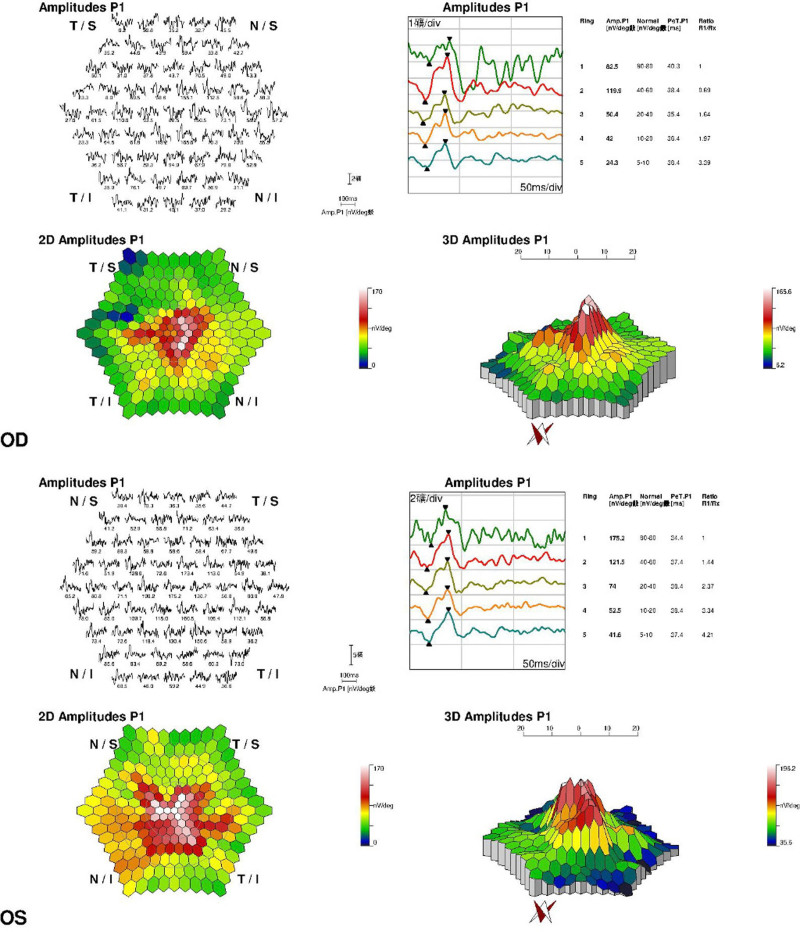
ERG showed impaired cone function in both eyes, with the left eye more severe damage than the right eye. ERG = electroretinogram.

Four months later, the patient returned to the ophthalmology clinic and reported a reduction in the extent of the visual field defect compared to the previous time. His visual acuity and intraocular pressure were unchanged as in the initial exam, and with normal anterior segments. Color fundus photography revealed reduced papilledema in both eyes compared to previous examinations. The patchy superficial hemorrhage in the macular region of the left eye was completely resolved, and the patchy superficial hemorrhage on the nasal side of the optic disc of the left eye was also significantly absorbed (Fig. 2A, B). OCT showed a reduction in papilledema (Fig. 2C, D). Humphrey’s visual field showed that the scotoma in the right eye has completely resolved (MD = +0.53dB), while the defect in the inferior nasal quadrant of the visual field in the left eye was smaller compared to its previous extent (MD = −7.75 dB; Fig. 2E, F). The ERG results demonstrated impaired cone function in both eyes, with a more pronounced deficit observed in the left eye compared to the right (Fig. 3).

## 3. Discussion

Previous reports have suggested that high-altitude and hypoxia as risk factors for HAR.^[5]^ Hypoxia can result in incomplete oxidation of the body’s tissues, leading to the accumulation of metabolites that exert toxic effects on the tissues. Consequently, this increases vascular permeability and gives rise to retinal edema, hemorrhage, and exudation.^[6]^ Weidman and Tabin classified HAR into 4 grades. Grade 1: Mild retinal hemorrhage of the retinal vein in 1 PD area; Grade 2: Moderately dilated retinal vein with retinal hemorrhage in 2 PD areas; Grade 3: Significantly dilated retinal vein with retinal hemorrhage in 3 PD areas, as well as perimacular retinal hemorrhage and vitreous hemorrhage; Grade 4: The retinal vein is significantly dilated and bluish-purple, the retinal hemorrhage is larger than in 3 PD areas, and the macula and retinal vein are tortuous and dilated, affecting central visual acuity.^[7]^ Meanwhile, high-altitude climbers often exhibit elevated levels of hematocrit and hemoglobin concentrations, which can result in heightened blood viscosity, increased clotting propensity, and diminished oxygen delivery.^[8]^ HAR was first described by Singh et al,^[9]^ who noted retinal vasculature changes and papilledema in 24 out of the 1925 patients (1.3%) diagnosed with acute mountain sickness occurring between 11,000 ft (3353 m) and 18,000 ft (5486 m) above mean sea level. In addition, Frayser et al^[10]^ found that 9 out of 25 adults (36%) had retinal hemorrhage at an altitude of 17,500 ft (5334 m). These are the earliest documented cases of HAR. Subsequent reports of related cases are rare.

Interestingly, Arora et al^[8]^ discovered that HAR is more prevalent among young, well-conditioned individuals at high altitudes. This may be due to dilation of blood vessels and an increase in systemic circulatory pressure, making the retina in young adults more susceptible to damage during physical exertion. It also indicates that young and middle-aged individuals are more prone to HAR under continuous high-intensity physical activity, which may further support the aforementioned statement. The study by Tian et al^[3]^ showed that 3 months after people returned to baseline altitude, their retinal condition could recover to normal, indicating that increased atmospheric oxygen content and reduced altitude restore nerve and vascular function. This could also provide evidence that the disease has a generally good prognosis. The occurrence and development of high-altitude sickness depends not only on the height of ascent, but also on the rate of ascent, adaptation to altitude, individual baseline levels, and other possible genetic and environmental factors.^[11]^ Our case reports that HAR occurred due to the individual’s body rapidly ascending from low altitude to high altitude within a short period of time, resulting in body struggling to immediately adapt to the high altitude and hypoxia. Therefore, the prevention is of utmost importance, with a particular emphasis on maintaining slow ascent rates and ensuring sufficient adaptation time.

After experiencing symptoms, the patient in this case failed to promptly supplement oxygen and gradually descend to a lower altitude. Consequently, the ocular pathology experienced only partial improvement, without achieving complete self-recovery. Once HAR is present, further ascent is stopped and oxygen supplementation and gradual descent to a lower altitude are recommended as the best treatment options.^[7]^ Other related reports showed that individuals can gradually develop tolerance to high-altitude and hypoxia during the process of slow acclimatization.^[11]^ Therefore, a climber who is already well adapted to such extreme conditions can ascend the Himalayas, reach (and subsequently descend from) the summit without the need for supplemental oxygen. This suggests the advantage of adopting a slow ascent rate and gradually adapting to hypoxia in preventing HAR. Moreover, Wiedman and Tabin observed 40 climbers at altitudes above 4870 m and found that all those with HACE had HAR.^[7]^ They argued that treatment of HACE with oxygen, steroids or diuretics should be initiated as soon as possible following a diagnosis of HAR, and that altitude reduction should be initiated immediately to prevent further development of HACE. Another study also confirmed that the severity of HAR was significantly associated with diseases such as HAPE and HACE.^[8]^ All the above studies demonstrate the importance of early diagnosis of HAR in preventing the development of life-threatening HACE.

As science progresses and examination techniques advance, early HAR can be identified and diagnosed. In addition to fundus photography and OCT, we also observed changes in the patient’s FFA, Humphrey’s visual field and ERG. The FFA revealed fluorescein leakage from the optic disc and obscuration of fluorescence due to retinal hemorrhage, which are typical manifestations of optic nerve and retinal vascular involvement in HAR. We additionally noted his visual field defect, and observed that his visual field defect did not fully recover to normal due to he fails to promptly leave the pathogenic environment. It is worth mentioning that ERG can be used for early diagnosis of HAR, and in this case, ERG was observed to detect impaired cone cell function in the early stages of HAR.

## 4. Limitation

There are limitations to this case report. While this case provides an in-depth perspective, it does not represent the full extent of the HAR risk faced by all high-altitude residents or individuals rapidly ascending to high altitudes. Environmental factors at high altitudes are complex, and in addition to hypoxia, low temperatures and intense UV radiation may also be related factors for HAR.

## 5. Conclusions

This case comprehensively reveals the clinical features of HAR, emphasizing that failure to leave the pathogenic environment in time may lead to permanent visual impairment. More importantly, this case highlights the critical importance of early prevention, diagnosis and prompt treatment of HAR to achieve better prognoses for patients.

## Author contributions

**Data curation:** Rao Fu, Yenan Wang.

**Formal analysis:** Rao Fu.

**Funding acquisition:** Dachuan Liu.

**Resources:** Rao Fu.

**Supervision:** Dachuan Liu.

**Visualization:** Rao Fu.

**Writing – original draft:** Rao Fu, Yenan Wang.

**Writing – review & editing:** Rao Fu, Yenan Wang, Dachuan Liu.
